# Optogenetic therapeutic strategies for diabetes mellitus

**DOI:** 10.1111/1753-0407.13557

**Published:** 2024-05-16

**Authors:** Xin Deng, Dandan Peng, Yuanfa Yao, Ke Huang, Jinling Wang, Zhihao Ma, Junfen Fu, Yingke Xu

**Affiliations:** ^1^ Department of Endocrinology Children's Hospital of Zhejiang University School of Medicine, National Clinical Research Center for Child Health Hangzhou China; ^2^ Department of Biomedical Engineering, MOE Key Laboratory of Biomedical Engineering, Zhejiang Provincial Key Laboratory of Cardio‐Cerebral Vascular Detection Technology and Medicinal Effectiveness Appraisal, Zhejiang Provincial Key Laboratory of Traditional Chinese Medicine for Clinical Evaluation and Translational Research Zhejiang University Hangzhou China; ^3^ Binjiang Institute of Zhejiang University Hangzhou China

**Keywords:** cell therapy, diabetes mellitus, insulin, insulin resistance, Optogenetics

## Abstract

Diabetes mellitus (DM) is a common chronic disease affecting humans globally. It is characterized by abnormally elevated blood glucose levels due to the failure of insulin production or reduction of insulin sensitivity and functionality. Insulin and glucagon‐like peptide (GLP)‐1 replenishment or improvement of insulin resistance are the two major strategies to treat diabetes. Recently, optogenetics that uses genetically encoded light‐sensitive proteins to precisely control cell functions has been regarded as a novel therapeutic strategy for diabetes. Here, we summarize the latest development of optogenetics and its integration with synthetic biology approaches to produce light‐responsive cells for insulin/GLP‐1 production, amelioration of insulin resistance and neuromodulation of insulin secretion. In addition, we introduce the development of cell encapsulation and delivery methods and smart bioelectronic devices for the in vivo application of optogenetics‐based cell therapy in diabetes. The remaining challenges for optogenetics‐based cell therapy in the clinical translational study are also discussed.

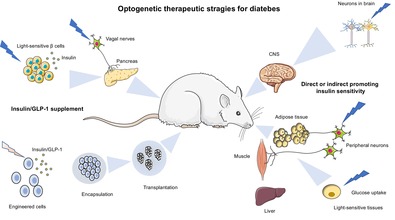

## Graphical abstract

Highlights

- Optogenetics has become a new evolving therapeutic approach for diabetes.
- Synthetic cells with light‐responsive modules for insulin/glucagon‐like peptide‐1 production have been developed to treat diabetes.
- Insulin‐responsive tissues are targets of optogenetics, which works to lower blood glucose and alleviate insulin resistance.
- The latest development of optogenetics in diabetes treatment and the remaining challenges are summarized.

## 1 INTRODUCTION

Diabetes mellitus (DM) and the associated complications are metabolic diseases with high morbidity that result in poor quality of health and life.^1^, ^2^ The main subtypes of DM are type 1 diabetes Mellitus (T1DM) and type 2 diabetes mellitus (T2DM), which are characterized by failure in insulin production and relative insulin deficiency mediated by autoimmune and metabolic mechanisms, respectively. Thus exogenous insulin administration, enhancement of insulin secretion, and improvement of dysfunctions in insulin signaling such as insulin resistance serve as the main approaches for diabetic therapy.^3^, ^4^, ^5^, ^6^, ^7^ Importantly, glucagon‐like peptide‐1 (GLP‐1) has the capacity to lower blood glucose independently of insulin secretion. Currently, innovative development that incorporates continuous glucose monitors into insulin pumps achieved algorithm‐driven automation of insulin delivery, which results in a significant reduction in the morbidity and mortality of DM.^8^, ^9^, ^10^ However, several considerable challenges remain, such as poor compliance, lifelong wearing or management, and invasive operation, which prevent patients from fulfilling the treatment goals.^11^, ^12^

It has been shown that alleviation of the dysfunctional β‐cells is a practical strategy for diabetic treatment, but the applications of whole‐organ and islet transplantation have been severely hampered by the shortage of suitable donors, which are gold‐standard procedures in control of glucose for diabetic patients.^13^ Although the production of functional β‐cells via chemical cocktails displays a promising future,^13^, ^14^ the protocols for cell differentiation are complicated, expensive, and likely incompatible with large‐scale bioprocessing. Additionally, differentiation of β‐like cells in vitro requires considerable time before implantation, which may limit the life span of the implant before its inactivation by fibrosis^15^ and lead to ex‐post risks such as carcinogenesis and hormonal disorders.^14^ Hence, the engineered cells based on synthetic biology and optogenetics could provide an attractive solution.

Synthetic biology has charted many notable achievements since it emerged.^16^ In therapeutic aspects, it has been used to design complex genetic networks for regulation of cellular activity, metabolism, and gene targets. Combined with efficient encapsulation technologies for engineered cell delivery, it exhibits therapeutic value for various human diseases in animal models.^17^, ^18^ Moreover, the incorporation of less invasive optogenetics into synthetic biology imparts the engineered cells with instantaneous regulation capability instead of time‐consuming feedback regulation and conditional or inducible gene expression. Besides, characteristics such as higher spatiotemporal resolution and reversibility permit more precise manipulation of cellular activities, which have achieved impressive methodological progress in neuroscience and other biological systems.^19^, ^20^, ^21^ Furthermore, optogenetics‐based technologies allow for remote and noninvasive controlling of immense therapeutic outputs, which have been widely applied to treat neurological diseases, diabetes, heart diseases, and cancer.^22^ Very recently, gene therapy and optogenetics have been applied in clinics for a fundamental improvement of vision and hearing restoration in patients. For example, clinical trials targeting the retinal ganglion cells to reinstate parallel retinal processing have shown to partially restore visual function in genetically induced blind patients.^23^ In this review, we summarize the latest advances in using optogenetics‐based synthetic biology approach for treating diabetes and its related complications, which include engineering β‐cells or non‐β‐cells for insulin or GLP‐1 production, enhancement of insulin sensitivity for glucose disposal, the delivery strategies for engineered cell therapy, as well as discussing the challenges in optogenetics‐based therapies for DM.

## 2 OVERVIEW OF OPTOGENETICS

Optogenetics, which combines optical and genetic methods, provides an unprecedented ability to use light for precise control of various cellular activities.^24^, ^25^ Optogenetic molecules change their protein conformation in response to light stimulation at specific wavelengths, which are currently found in all kingdoms of life, including plants, bacteria, and animals.^26^ Optogenetic tools take advantage of light modulation with fast “on–off” kinetics, tunable excitation, and emission wavelengths, which can noninvasively regulate cellular targets with exquisite spatial and temporal resolution.^27^

Upon light stimulation, most photosensors harbor chromophores that would covalently or noncovalently bind to apoproteins. Photoinduced conformational changes on chromophores induce structural changes of these apoproteins, which then trigger corresponding downstream signaling pathways.^28^ Photoreceptors have been classified into distinct families based on different chromophores (the common optogenetic modules are listed in Table 1). Rhodopsin family proteins incorporate retinal as a chromophore and are further classified into two groups, namely microbial (type I) and animal (type II) opsins. Animal rhodopsins are G‐protein‐coupled receptors (eg, melanopsin), but the functions of microbial rhodopsins are very divergent such as ion pump (halorhodopsin), ion channel (halorhodopsins, ChR2), and signaling/enzyme rhodopsins (guanylyl cyclase).^29^ The second family shares the same cofactor, the blue‐light sensitive flavin chromophore. They include cryptochromes (CRYs), light‐oxygen‐voltage (LOV)‐domain proteins (eg, blue‐light photoreceptor Vivid [VVD]), and blue light usingflavin adenine dinucleotide‐domain proteins (eg, *Beggiatoa* photoactivated adenylyl cyclase [bPAC], and red/near‐infrared‐light‐regulated diguanylate cyclase [BphS]).^30^ These blue‐light‐sensitive photoreceptors are most versatile for optogenetics.^31^ The third family, the linear tetrapyrrole‐binding photoreceptors, include phytochromes and cyanobacteriochromes. They can be activated by far‐red light that has a deeper penetration depth.^30^ Other optogenetic modules include cobalamin (vitamin B12) binding domains (CBDs) of bacterial CarH transcription, xanthopsins, and orange carotenoid protein, which use 5′‐deoxyadenosylcobalamin chromophore, p‐coumaric acid, and ketocarotenoid as their corresponding cofactors.^32^, ^33^, ^34^

**TABLE 1:** The common optogenetic modules and their photophysical properties.

Family	Light‐sensitive proteins	Photoreceptors	Functions	Activation *λ* (nm)	Deactivation *λ* (nm)	Association time	Dissociation time
Rhodopsin	Microbial (type I) opsins	ChR2	Cation channel	450	Dark	Seconds	Minutes
	Animal (type II) opsins	Melanopsin	G‐protein coupled receptors (GPCRs)	500	Dark	Seconds	Seconds
Blue‐light sensitive flavin chromophore	Cryptochromes (CRYs)	CRY2‐CIBN	Hetero‐dimerization	450	Dark	Seconds	Minutes
	Light‐oxygen‐voltage (LOV)	VVD	Homo‐dimerization	450	Dark	Seconds	Minutes
	FAD (BLUF)‐domain	bPAC	Production of cAMP	450	Dark	Seconds	Seconds
	proteins	BphPS	Production of c‐di‐GMP	450	Dark	Seconds	Seconds
Linear tetrapyrrole‐binding photoreceptors	Phytochromes (Phys)	Biliverdin Ixα	Hetero‐dimerization	660	730	Seconds	Seconds
	Cyanobacteriochromes (CBCRs)	Biliverdin Ixα	Autophosphorylation	334–672^a^	Dark	Seconds	Minutes

Abbreviations: bPAC, *Beggiatoa* photoactivated adenylyl cyclase; BphPS, bacteriophytochrome photoreceptor; cAMP, cyclic adenosine monophosphate; c‐di‐GMP, cyclic diguanylate; ChR2, channelrhodopsin‐2; FAD‐BLUF, blue light using flavin adenine dinucleotide‐domain proteins; VVD, Vivid.

^a^ The CBCRs show extensive diversity in the spectral property and mostly are stable under dark conditions. Functions refer to the modes of action for the photoreceptors. Activation and deactivation *λ* (nm) means the optimal wavelengths or conditions for controlling the activity of the light‐sensitive proteins. Association and dissociation time refer to the half times of protein activation and its recovery respectively.

The light‐induced conformational change of photoreceptors could be classified as heterodimerization, homodimerization, clustering, uncaging, and dissociation.^26^ Optogenetics‐induced protein heterodimerization and homodimerization employ several photosensitive domains, such as the derivatives of LOV domains, cryptochromes (CRY/CIB), phytochromes, and ultraviolet response locus 8. The construction of these proteins and their corresponding binding partners with specific cellular targeting motifs would allow for activation of signaling propagation at specific locations.^35^ Moreover, the light‐controlled protein clustering systems such as CRY2 (E490G) mutant has been used to induce protein phase condensation or develop synthetic membrane‐less organelles to regulate metabolic enzyme activities.^36^ Some photoreceptors can be structurally switched from open or closed conformation, which have been used to expose a protein motif of interest, such as a nuclear localization signal (NLS) or nuclear export signal (NES) to regulate protein localization with light.^31^, ^37^ For example, by fusion of the NES peptide to the Jα helix of the AsLOV2 domain, it could act as an allosteric photoswitch that allows for nuclear exports of protein of interest.^38^ Additionally, photodissociable systems such as photodissociable Dronpa and CBDs dissociate into monomers upon violet and green light exposure respectively,^39^, ^40^ which also have fascinating applications in regulation of certain biological processes. Collectively, by sophisticatedly designing and choosing the appropriate photoreceptors, we can use light to control intracellular activities or treat diseases by modulating the function of the protein of interest and its subcellular location, or by light‐induced gene expression.^41^

## 3 OPTOGENETICS‐BASED ENGINEERED CELL FOR INSULIN AND GLP‐1 PRODUCTION

### 3.1 Molecular mechanisms of insulin secretion

**FIGURE 1:**
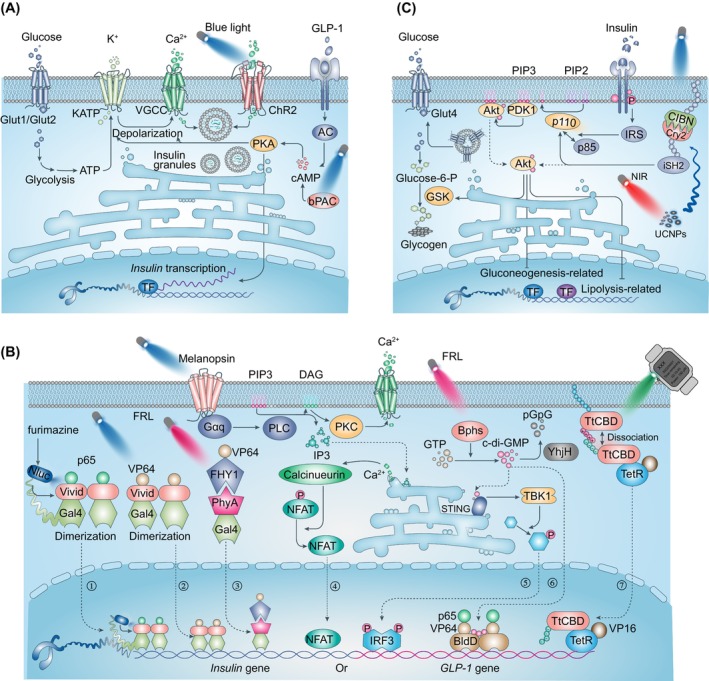
Optogenetics‐based engineered cells for insulin and GLP‐1 production. (A) Left: Glucose enters β cell through the glucose transporter protein 1 or 2 (GLUT1/GLUT2) and undergoes glycolysis to generate adenosine triphosphate (ATP). The elevated cytosolic ATP leads to the closure of ATP‐sensitive potassium (KATP) channels, which induce membrane depolarization and opening of the VGCC. The increase of intracellular calcium promotes insulin granule exocytosis in β cells. Right: Optogenetic‐controlling of second messengers to promote insulin secretion in β cells. Blue light stimulates channelrhodopsin‐2 (ChR2) or photoactivates adenylyl cyclase (bPAC), which causes calcium influx and cAMP production respectively to promote insulin secretion. (B) Optogenetics‐based gene expression in noninsulin secretion cells. ①②: Upon blue‐light activation, the protein vivid changes its structure to induce the dimerization of the hybrid transactivator. It consists of the Gal4 DNA binding domain, the photosensitive protein vivid, and a VP16 or p65 transactivation domain binding to the upstream activating sequence of Gal (UASG) sequence to initiate gene transcription. Fusing NanoLuc (Nluc) luciferase to this hybrid transactivator allows for using furimazine (luciferase substrate) and blue light to initiate the transcription of the gene of interest. ③: The Δphytochrome A (ΔPhyA)‐based photoswitch (REDMAP) system was designed by fusion of a truncated PhyA variant (amino acids 1–617) to the yeast Gal4 and linking FHY1 (amino acids 1–202) to transactivator VP64, respectively. Under pulsed red‐light illumination, the activated PhyA binds to FHY1‐VP64 and through VP64 recruits transcription factors to initiate insulin expression. ④: Blue light stimulates melanopsin, which sequentially activates the Gaq‐type G protein (Gaq), phospholipase C (PLC), and phosphokinase C (PKC), leading to elevated intracellular Ca^2+^ levels by activation of transient receptor potential channels (TRPCs) and endoplasmic reticulum (ER) activity. The elevated calcium activates the nuclear factor of activated T‐cells (NFAT) via the calcium sensor protein calmodulin, which triggers the synthetic promoter (PNFAT) to drive the downstream gene *GLP‐1* expression. ⑤⑥: The far‐red light (FRL)‐controlled engineered cell systems. FRL (~730 nm) activates the photoreceptor BphS, which converts guanylate triphosphate (GTP) into c‐di‐GMP and intracellular c‐di‐GMP can be degraded by c‐di‐GMP–specific phosphodiesterase (YhjH). ⑤ was based on the endogenous human STING pathway, where c‐di‐GMP activates STING and triggers tank‐binding kinase 1 (TBK1), resulting in nuclear translocation of phosphorylated interferon regulatory factor 3 (IRF3) and subsequently gene regulation. ⑥ was designed with synthetic transactivators consisting of BldD (c‐di‐GMP‐binding domain), p65, and VP64 transactivation domain. The c‐di‐GMP triggers BldD dimerization and binding to the promoter to regulate gene expression. ⑦: A membrane‐tethered TtCBD combines with a synthetic cytosolic TtCBD‐TetR‐VP16 transactivator fusion protein. The TtCBDs dissociate and release the transcription factor, which activates the expression of transgenes upon smart‐watch‐programmed green light illumination. (C) Optogenetic control of insulin signaling. Left: The binding of insulin to its receptor initiates insulin‐receptor substrate (IRS) protein phosphorylation, which recruits the p85 regulatory subunits of p85/p110 PI‐3K kinase to the cell membrane. This promotes the conversion of PtdIns(4,5)P_2_  (PIP2) to the phospholipid phosphatidyl 3,4,5‐phosphate (PIP3) on the cell membrane, facilitating the recruitment and subsequent activation of the protein kinases PDK1 and Akt. Insulin promotes the translocation of glucose transporter protein GLUT4 from intracellular vesicles to the plasma membrane, facilitates glycogen synthesis, and suppresses gluconeogenesis and lipolysis. Right: Upconversion nanoparticles (UCNPs) transduce near‐infrared (NIR) light to visible blue light to promote the membrane translocation of mCherry‐CRY2‐iSH2 for its binding with CIBN‐CAAX, which activates PI3K/AKT signaling pathway for the optogenetics‐based insulin activation and glucose disposal. AC, adenylate cyclase; cAMP, cyclic adenosine monophosphate; C‐di‐GMP, cyclic diguanylate; CIBN DAG, diacylglycerol; FHY, far‐red elongated hypocotyl 1; GLP‐1, glucagon‐like peptide‐1; GSK, glycogen synthase kinase; PDK, pyruvate dehydrogenase kinase 1; PIP3, phosphatidylinositol‐3,4,5‐triphosphate; TetR, tetracycline‐dependent receptor; TF, transcription factor; BphS, red/near‐infrared‐light‐regulated diguanylate cyclase; CIBN, the N‐terminal variant of cryptochrome‐interacting basic helix‐loop‐helix protein; CRY2, cryptochrome 2; STING, the stimulator of interferon genes; TtCBD, Thermus thermophilus cobalamin binding domain.

Insulin is a unique glucose‐lowering hormone that is secreted by the pancreatic β cells. The β cells secrete insulin in a biphasic manner after glucose stimulation (Figure 1A). The glucose uptake in pancreatic β cells is mediated by the glucose transporter 1 in humans or by glucose transporter 2 in rodents. The uptaken glucose is then utilized by aerobic glycolysis and mitochondrial metabolism. The concomitant adenosine triphosphate (ATP) generation increases the ATP/adenosine‐diphosphate ratio, leading to the closure of K_ATP_‐channels and the opening of voltage‐gated calcium channels (VGCC) at the cell surface, which eventually triggers insulin granule exocytosis.^42^ In addition, multiple metabolic signalings also contribute to the amplification of insulin secretion. For instance, the cyclic adenosine monophosphate (cAMP) is biosynthesized by adenylate cyclase in response to hormone stimulation, such as GLP‐1 and glucose‐dependent insulinotropic peptide (GIP),^43^ which has been shown to augment insulin secretion.

### 3.2 Design and development of light‐sensitive β cells for glucose control

The engineered modifications of β cells are mostly targeted on the key molecules in glucose‐regulated insulin secretion signaling pathway, or more precisely, on optogenetic control of calcium,^44^ cAMP,^45^ and cyclic diguanylate (c‐di‐GMP) activities^46^ (Figure 1A). It has been shown that infection of the mouse pancreatic β‐cell line with ChR2 (light‐gated cation channel) lentivirus conferred a light‐regulated insulin secretion.^44^ The light‐induced ChR2 activation stimulates Ca^2+^ uptake and depolarizes β cells, which results in promotion of insulin secretion.^44^ The transplantation of ChR2‐expressing β cells in T1DM mice has been shown to improve the oral glucose tolerance test (OGTT) and alleviate glucose metabolism. Furthermore, transgenic mice with ChR2 especially expressed in islets were shown to increase calcium influx and insulin release in high‐fat diet‐induced animals after light stimulation.^47^ Interestingly, similar to photosensitive proteins, a photoswitchable sulfonylurea, JB253, has been shown to block K_ATP_ channel activity upon violet‐blue light illumination,^48^ which has been successfully applied to regulate insulin secretion in pancreatic β cells. Recently, another group attempted to optogenetically control insulin secretion from pancreatic islet‐like organoids derived from human pluripotent stem cells, through which to eliminate the risk of immunogenicity or genotoxicity in clinical applications. In this study, the optogenetic module monSTIM1 (monster‐opto‐stromal interaction molecule 1) was developed to induce Ca^2+^ transients thus to promote insulin secretion.^49^

These optogenetic systems rely on light stimulation to trigger β cell insulin secretion, which are irrelevant on glucose‐related signaling. In some circumstances, the leakage of light would induce considerable amounts of insulin release from the engineered β‐cells, which would cause hypoglycemia. Therefore, the engineered β‐cells that are capable of regulating the cAMP levels by light were developed. The cellular cAMP levels are essential for both neurohormone‐mediated and glucose‐stimulated insulin release. Moreover, as a physiological amplifier of glucose‐stimulated insulin secretion (GSIS), different from Ca^2+^, light‐induced cAMP production does not promote the release of insulin without glucose.^50^ bPAC has been shown to produce cAMP upon blue‐light illumination, which enables spatiotemporally manipulating intracellular cAMP levels by light.^51^ Zhang et al ectopically expressed bPAC in cultured β‐cells or murine islets, and the light‐induced elevation of intracellular cAMP was shown to promote GSIS. This system is also capable of regulating blood glucose in T1DM mice,^45^, ^52^ and as expected, increasing the intracellular cAMP with photoactivation of bPAC has limited function on GSIS without glucose.

### 3.3 Optogenetic control of insulin or GLP‐1 production in noninsulin secretion cells

Engineering of non‐β‐cells overcomes the absence of suitable donor cells or tissue, which is a time‐saving approach for diabetic cell therapy. In theory, this strategy requires an applicable sensor to monitor the postprandial blood glucose increase and sensitive feedback regulation loop of insulin or insulinotropic protein expression. In optogenetics‐based diabetic therapy, photosensitive proteins act as semiautomatic glucose monitors that could be externally triggered by light when patients have a meal and function as upstream regulators of insulin or insulinotropic protein expression. Two strategies have been successfully applied to construct optogenetics‐based gene switches for protein production: (a) photoactivation of signaling pathways to recruit or activate transcription factors^53^; and (b) light‐triggered dimerization of modified DNA‐binding protein and transactivator protein or homodimerization of a modified hybrid transactivator for target gene expression.^54^

A previous study by Ye et al demonstrated the optogenetics‐based diabetic therapy that used blue light to optically regulate phospholipase C/inositol triphosphate/calcium ion pathway via melanopsin, a light‐sensitive G protein‐coupled receptor (GPCR). They employed a calcium‐responsive transcription factor, namely the nuclear factor of activated T‐cells (NFAT), to initiate *GLP‐1* gene expression in HEK‐293 cells (Figure 1B.④). The light‐induced increase in intracellular calcium would indirectly promote NFAT activation and thus induce GLP‐1 expression.^53^ These engineered HEK‐293 cells were microencapsulated and implanted into T2DM mice, and improved blood glucose homeostasis was detected by upregulation of GLP‐1 expression after continuous light illumination.

However, these optogenetic modules exhibit slow activation/deactivation kinetics and poor tissue penetration. To overcome these drawbacks, the same group designed another optogenetic tool, a red/far‐red light‐mediated and miniaturized Δphytochrome A (ΔPhyA)‐based photoswitch (REDMAP) system, which is established on the PhyA/far‐red elongated hypocotyl 1 (FHY1) optogenetic elements^55^ (Figure 1B.③). Photosensitive protein PhyA binds to its partner FHY1 in the presence of red‐light (660 nm) stimulation and disassociates from each other upon far‐red light (730 nm) illumination. The REDMAP system was designed by fusion of a truncated PhyA variant (amino acids 1–617) to the yeast Gal4 transcription factor (amino acids 2–146 including DNA‐binding domain and homodimerization domain) and linking FHY1 (amino acids 1–202) to transactivator VP64, respectively. The hybrid DNA‐binding protein (PhyA–Gal4) can bind to the yeast upstream activating sequence (Gal4 binding site) upstream of minimal human cytomegalovirus insulin sequence. Under pulsed red‐light illumination, the activated PhyA binds to FHY1‐VP64 and through VP64 recruits transcription factors to initiate insulin expression. The implantation of these engineered HEK‐293 cells has been demonstrated to significantly elevate serum insulin and GTT showed a significant improvement of glucose homeostasis in both T1D mice and T1D rats after illumination. In addition, another optogenetic tool, Bphs, was developed, which is excited by red light and has been shown to biosynthesize c‐di‐GMP in response to light illumination. Ye et al expressed Bphs in HEK‐293 cells and Bphs indirectly recruited transcription factor interferon regulatory factor 3 via STING/TANK‐binding kinase 1 and activate a chimeric transactivator FRTA3 (p65‐VP64‐NLS‐BldD [c‐di‐GMP effector protein]) via its product c‐di‐GMP^46^ (Figure 1B.⑤⑥). Moreover, when these engineered HEK‐293 cells or human mesenchymal stem cells^56^ were implanted into diabetic mice, light‐induced insulin or GLP‐1 gene expression by activation of these transcription factors is sufficient for controlling the blood‐glucose homeostasis.^46^

As dimerization is essential for Gal4‐based gene regulation, studies have used light‐induced protein homodimerization systems, such as the LOV‐based system VVD, to artificially induce Gal4 cluster. For example, Wang et al coexpressed Gal4 and transactivator VP16 by VVD element, which rapidly binds to the promoters upon blue‐light exposure and initiates transcription of targeted genes in cells (Figure 1B.②). They used this approach to promote insulin expression in diabetic mice and to regulate blood glucose homeostasis.^57^ Recently, Mansouri et al developed a traceless controlled mammalian gene switch, which is composed of an engineered membrane‐tethered light‐sensitive cobalamin‐binding domain of Thermus thermophilus (TtCBD) of CarH protein and a synthetic cytosolic TtCBD‐transactivator fusion protein.^58^ The TtCBDs dissociate and release the transcription factor, which activates transgenes expression upon light illumination (Figure 1B.⑦). As TtCBDs constitutively assemble as a homo‐oligomer, they equipped the membrane‐anchored Myr‐TtCBD and TtCBD‐TA with positively or negatively supercharged domains, which increases the specificity of transdimer interaction. Studies have shown that the engineered cells can effectively treat T2DM mice by producing and releasing shGLP‐1.^58^ Moreover, a toolbox that induces glucose‐dependent of insulin secretion has been developed, which is functional only in the presence of both high glucose and blue‐light illumination. The high blood glucose induces the expression of the DNA binding domain of the transcription factor Gal4 by using glucose‐sensitive GIP promoter, whereas the blue light controls the translocation of transactivation domain VP16 to the promoter of insulin to induce protein expression.^59^

All aforementioned optogenetic elements are activated with visible light that has limited penetration in tissues and animals.^60^ Therefore, micro‐LED implantation^61^, ^62^ and injection of upconversion nanoparticles (UCNPs) that converts near‐infrared light (NIR) into visible light spectrum are becoming possible.^63^, ^64^, ^65^ Besides, there is another possibility that uses bioluminescence to activate optogenetic modules, the BRET‐activated optogenetics system.^66^ Recently, Li et al reported a LuminON system that harbors a nano luciferase (Nluc) as the endogenous light source and a light‐switchable transcription factor.^67^ They constructed and screened a Nluc‐Gal4‐VVD‐p65 transcription factor and injected luciferin to activate Nluc for light stimulation of VVD (Figure 1B.①). Then, the engineered cells expressing the LuminON system, which can stably produce insulin, were intraperitoneally implanted into T1DM mice. The blood‐glucose homeostasis and OGTT could be regulated by the luciferin dosage.^68^ Additionally, Nluc also acted as a gene expression reporter in melanopsin‐mediated optogenetic control of glucose homeostasis in T1DM animals.^69^ This study expressed melanopsin into a β cell‐like cell line (INSvesc cell line, a variant of the established pancreatic β‐cell line 1.1E7) with the capability of insulin secretion in response to calcium influx. They modified the proinsulin sequence by replacing C peptide with Nluc, and with that light illuminated membranal melanopsin would induce calcium uptake and subsequently trigger insulin exocytosis. Importantly, they overcame an inevitable delay in insulin secretion and developed specialized illumination equipment. Collectively, bioluminescence can serve as another valuable tool for controllable cell therapy in diabetes.^68^ This therapeutic strategy could activate deeper engineered cells or cell implantation, which is comparable to drug‐induced gene expression systems. Collectively, these studies capitalize on different optogenetic systems to control cellular activities for producing insulin in β cells or producing insulin and GLP‐1 in non‐β cells, which have demonstrated beneficial effects of DM therapy (Figure 1 and Table 2).

**TABLE 2:** Summary of the optogenetics‐based cell therapies in treatment of diabetes.

	Optogenetic modules	Sites of action	Cell types	Animals	Diabetes‐related improvements	Light illumination conditions	References
β cells	ChR2	Ca2+‐dependent insulin secretion	MIN6; cell transplantation	T1DM	IPGTT; Glucose greatly decreased in 30 min	10 and 20 s (18.6 and 19.1 mM, respectively)	Kushibiki et al 2015^44^
	ChR2	Ca2+‐dependent insulin secretion	Intact islets, transgenic ChR2 mice	HFD	IPGTT; insulin: 5‐fold increase	1 h constant light stimulation	Reinbothe et al 2014^47^
	JB253, JB558	K+‐dependent insulin secretion	Human and mouse islets	‐	Insulin: 4–8 fold increase	30 s ON /90 s OFF for 1 h	Broichhagen et al. 2014^48^
	bPAC	cAMP‐dependent insulin secretion	MIN6 and murine islets	‐	cAMP: 6–8‐fold; insulin: ~3.5‐fold	0.18 mW/mm^2^; 5 s ON /10 s OFF for 30 min	Zhang et al 2017^52^
	bPAC	cAMP‐dependent insulin secretion	MIN6; cell transplantation	T1DM	GTT: improved; reduction of glucose after 2 days of transplantation	30 min on/off for 2–5 days after transplantation	Zhang et al 2019^45^
	monSTIM1	Ca^2+^‐dependent insulin secretion	Pancreatic islet‐like organoids; transplantation	T1DM	GTT: improved; c‐peptide and insulin: 1–2 fold	1 mW/cm^2^ for 2 h	Choi et al 2023^49^
non‐β cells	Melanopsin	GPCR/NFAT pathway‐dependent GLP‐1 expression	HEK‐293; microencapsulated	T2DM mice	GTT: improved	1.5 × 10^19^ photons s^−1^ m^−2^ 5 s ON/10 s OFF for 48 h	Ye et al 2011^53^
	PhyA/FHY1	Dimerization Gal4‐PhyA/FHY1‐VP64 for insulin expression c‐di‐GMP/p65‐VP64‐BldD pathway‐dependent insulin and	HEK‐293; microencapsulated, alginate‐poly‐(l‐lysine)‐alginate	T1DM mice and rats	Insulin, IPGTT: improved	Long‐term: (20 mW/cm^2^) for 2 days (1 min/day for mice; 5 min/day twice for rats and rabbits	Zhou et al 2021^55^
	BphPS	GLP‐1 expression	HEK‐293; microencapsulated, alginate‐poly‐(l‐lysine)‐alginate	T2DM db/db and T1DM mice	Rapid restoration of glucose; GTT, IR: improved	25 mW/cm^2^, 4 h/day for 15 days	Shao et al 2017^46^
		Homodimerization Gal4‐vivid‐VP64 for insulin expression					
	VVD	Furimazine or light‐induced homo‐dimerization of Nluc‐Gal4‐VVD‐p65 to initiate insulin expression	HEK‐293; hydrodynamic transfection liver	T1DM mice	Rapid restoration of glucose	90 mW/cm^2^ for 8 h	Wang et al 2012^57^
	VVD	c‐di‐GMP/p65‐VP64‐BldD dependent insulin expression	HEK‐293; microencapsulated, alginate‐poly‐(l‐lysine)‐alginate	T1DM mice	Dose‐dependent blood‐glucose homeostasis; IPGTT: improved	Short‐term (3 h): 5 mg/kg furimazine; long‐term (13 days): 7 mg/kg furimazine per day	Li et al 2021,^68^
	BphPS	GPCR/Ca2+ pathway‐dependent insulin secretion	hMSC (human mesenchymal stem cells); microencapsulated	T1DM mice	Long‐term glucose control; alleviates oxidative stress and renal damage	10 mW/cm^2^, 2 h/day for 40 days	Yu et al 2022^56^
	Melanopsin	Relocation of TtCBD‐TetR‐VPR (transactivator) to the nucleus to induce GLP‐1 expression	iβ‐cells (a variant of the established pancreatic β‐cell line 1.1E7.)	T1DM mice	A high fold of insulin secretion within 15 min; IPGTT: improved	White light (2–5 mW/cm^2^) 15 min/day for 7 days	Mansouri et al 2021^58^
	TtCBD		HEK‐293 T; microencapsulated, alginate‐poly‐(l‐lysine)‐alginate	T2DM mice	FPG: lower; long‐term glucose control; IPGTT, IR: improved	Green light (150 μW/cm^2^, 30 s ON/30 s OFF) for 12 h daily	Mansouri et al 2021^69^

*Note*: The table summarizes the key studies published on optogenetics‐based cell therapies for treatment of diabetes. The optogenetic modules, their sites of action, the specific cell types or animals studied, the light illumination conditions used and their improvement in diabetes were also listed.

Abbreviations: bPAC, *Beggiatoa* photoactivated adenylyl cyclase; BphPS, bacteriophytochrome photoreceptor; cAMP, cyclic adenosine monophosphate; c‐di‐GMP, cyclic diguanylate; ChR2, channelrhodopsin‐2; FHY1, far‐red elongated hypocotyl 1; FPG, fasting plasma glucose; GLP‐1, glucagon‐like peptide‐1; GPCR, G protein‐coupled receptor; HFD, high‐fat diet; IPGTT, intraperitoneal glucose tolerance test; IR, insulin resistance; MIN6, mouse pancreatic β‐cell line; monSTIM1, monster‐opto‐stromal interaction molecule 1; NFAT, nuclear factor of activated T‐cells; Nluc, nano luciferase; PhyA, phytochrome A; T1DM, type 1 diabetes mellitus; TetR, tetracycline‐dependent receptor; TtCBD, Thermus thermophilus cobalamin binding domain VVD, Vivid.

## 4 OPTOGENETIC TECHNIQUES FOR AMELIORATION OF INSULIN RESISTANCE AND NEUROMODULATION OF INSULIN SECRETION

### 4.1 Optogenetic regulation of insulin signaling

In addition to stimulation of insulin or GLP‐1 secretion, approaches for improvement of insulin resistance are equally important. Insulin resistance refers to a decreased metabolic response to insulin in target cells, especially in skeletal muscle, adipose tissue, and liver. Insulin‐stimulated glucose uptake into muscle and adipose tissue is highly susceptible to insulin resistance, where the dysfunctions in the insulin signaling pathway play a key role in obesity‐associated insulin resistance and T2DM.^6^, ^70^ Therefore, amelioration of insulin resistance has become an important therapeutic target for diabetic treatment.

It has been well known that insulin binds to its receptor and the autophosphorylation of the receptor recruits insulin receptor substrates, which further activate the phosphoinositide 3‐kinase (PI3K)/Akt signaling and other downstream pathways. Yan et al optically modulated PI3K/Akt signaling via control of a PI3K activator iSH2 localization (CIBN‐CAAX and CRY2‐iSH2).^71^, ^72^ The blue‐light stimulation induced the recruitment of cytosolic localized CRY2‐iSH2 to the membrane where CIBN‐CAAX resides.^73^ Membrane targeting of iSH2 recruited endogenous PI3K by interaction with its catalytic subunit p110α, which further led to Akt phosphorylation activation (Figure 1C). After transfection of hepatic tissue with their system, they successfully used NIR to reduce blood glucose and OGTT in T2DM mice via upconversion nanoparticles.^72^ In obesity‐related T2DM, enhancement of fat‐burning metabolism such as adipocyte thermogenesis could indirectly benefit insulin resistance via increasing glucose consumption.^74^, ^75^ This action is primarily mediated by uncoupling protein 1 (UCP1)‐dependent pathway. Other studies support that non‐shivering heat generation by brown adipose tissue (BAT) is regulated by transmembrane GPCRs Opsin3 (Opn3) and Opsin 5 (Opn5). Light exposure to BAT increased thermogenic capacity by regulating fuel metabolism and mitochondrial respiration through Opn3‐GPCR,^76^ whereas Opn5, expressed in the mouse and primate hypothalamus, has been shown to inhibit violet light‐induced BAT thermogenesis.^77^ Recent research has demonstrated a noncanonical UCP1‐independent mechanism in beige fat, in which ATP‐dependent Ca^2+^ futile cycling may enhance adipose tissue thermogenesis. Tajima et al employed an implantable wireless micro‐LED device to stimulate beige fats with ChR2 expression, which results in Ca^2+^ cycling for noncanonical thermogenesis and protects mice against diet‐induced obesity.^78^ In addition, light‐sensitive melanopsin has been identified in human white adipose tissue, which might act as a peripheral circadian sensor, supporting the notion that lack of sufficient bodily exposure to sunlight may contribute to the current epidemics of obesity, diabetes, and cardiovascular disease. Therefore, these findings persuade us to investigate the function of light sensing in nonconventional photoreceptive cell types and investigate the potential molecular foundation for further evaluation of light‐based therapy.^79^ However, the mechanism of insulin resistance is far more complex than expected, and these studies are either limited to a certain tissue or directly regulate the signaling pathway, which needs more studies to prove their validity.

### 4.2 Optogenetic control of nervous activity for regulation of glucose homeostasis

It has been shown that the hypothalamus can indirectly control thermogenesis and β cell proliferation and function and motivate glucose‐seeking behavior by integration of various peripheral hormone secretion signals (eg, insulin, leptin, glucagon, and hormones from enteroendocrine cells) and control of both the sympathetic and parasympathetic activity.^80^, ^81^ In the hypothalamus, glucose‐sensing neurons are mainly localized in the arcuate, the ventromedial, and the lateral nuclei. For example, in arcuate neurons, proopiomelanocortin neurons activate and stimulate anorectic and catabolic responses via autonomic nervous system when sensing energy sufficiency (eg, stimulation from leptin and insulin). Conversely, signals of energy deprivation (eg, ghrelin and hypoglycemia) activate the agouti‐related peptide (AgRP) neurons and stimulate feeding and anabolic processes in peripheral tissues.^82^ This distinct regulation of feeding behaviors is crucial for maintaining glucose homeostasis and preventing the development of insulin resistance.^83^

Although the underlying mechanisms for regulatory roles of CNS in glucose homeostasis remain to be elucidated,^84^ several optogenetics‐based studies have contributed to dissecting the puzzles and have a potential therapeutic prospect. For example, the glucose‐sensing neurons in the ventromedial nuclei of the hypothalamus, especially those neurons expressing steroidogenic factor‐1 (SF‐1) and/or the gene encoding glucokinase, exert an effect on maintenance of energy balance and glucose metabolism. Optogenetic inhibition of the SF‐1‐expressing neurons impairs the recovery from insulin‐induced hypoglycemia, whereas optogenetic activation of those neurons increases blood glucose and induces profound hyperglycemia.^85^ It has been known that striatal dopamine is involved in regulation of energy homeostasis. Studies have tried to express ChR2 in dopamine D1 receptor (D1R)‐expressing neurons and used light to stimulate D1R positive neurons, which was demonstrated to enhance glucose tolerance in mice through the regulation of insulin sensitivity.^86^ Moreover, the extensive applications of optogenetics in neuroscience provide a powerful tool to explore complicated mechanisms of regulating blood glucose homeostasis in the brain. Steculorum et al used chemogenetic and optogenetic tools to acutely activate AgRP neurons and found systemic insulin sensitivity and glucose tolerance were impaired.^87^ Importantly, the activation of AgRP‐neuron in inhibition of insulin‐stimulated glucose uptake in BAT is partially exerted through the reprogramming of certain gene expression profiles toward myogenic signature.^87^

The secretion in the pancreas is under the regulation of neural modulation, which is exemplified by insulin secretion after the electrical stimulation of the vagus. Arjun et al used the choline acetyltransferase promoter to selectively express ChR2 into the parasympathetic axons (cholinergic cells).^88^ Interestingly, they found that light stimulation in different locations (abdominal cholinergic axons terminated within islets and the cervical vagus nerve) induced different effects in regulation of blood glucose, in which stimulation of the former location lowered blood glucose whereas the latter had no effect, and stimulation of both positions led to increased serum insulin.^88^ Similarly, another group expressed ChR2 into vagal nerves and implanted with UCNPs in the pancreatic ducts of the mice. The NIR light illumination activated the vagal nerves innervating pancreas, which enhanced GSIS and β‐cell proliferation.^89^ This approach might have advantages over β‐cell transplantation as it is less invasive and more physiological. In addition, light stimulation of the sympathetic nerves was shown to activate brown fat, which increased thermogenesis and the transcriptional regulation of *UCP1*.^75^, ^90^ This light‐regulated modulation of sympathetic nerves demonstrated that intracellular glycolysis and lactate shuttle are important for nonshivering thermogenesis.^91^ Vascular and neuronal regulation of islet blood flow has shown to modulate hormones secretion, and the acute activation of islet pericytes by ChR2 decreases islet blood flow, which in turn affects islet hormone secretion. This may partly explain the underlying mechanism between vascular alterations and islet dysfunction.^92^, ^93^

## 5 OPTOGENETIC THERAPEUTIC STRATEGIES AGAINST DIABETIC COMPLICATIONS

Diabetes‐induced chronic complications are very common, which can be divided into macrovascular complications such as cardiovascular disease, and microvascular complications that affect the kidney, retina, and nervous system.^94^ Although a large number of new treatments have been developed to improve diabetic complications in clinical trials, it is still hard to achieve satisfactory outcomes and meet the treatment goals.^95^ Among these complications, optogenetics has shed new light on treatment of diabetic retinopathy (DR), because it has been used to partially restore vision for retinal degenerative diseases. Recently, a study used blue light to activate the ChR2‐expressing cholinergic cells and observed a significant increase in the retinal capillary blood flow, which provides a potential strategy to mitigate blood flow impairment in the early stage of DR.^96^ Moreover, the implantation of aforementioned insulin‐secreting cells not only achieves long‐term blood glucose control but also alleviates oxidative stress and renal damage in diabetic mice.^56^ Management of hyperglycemia in renal dysfunction, especially those accompanied by decreased glomerular filtration rate, is challenging.^97^ However, studies have demonstrated that optogenetics‐based cell therapy can be applied to improve renal function (eg, serum creatinine, blood urea nitrogen, urine protein), and alleviate pathological damages (eg, collagen accumulation, thickening of the glomerular basement membrane).^56^

## 6 STRATEGIES FOR ENGINEERED CELL ENCAPSULATION AND DELIVERY

The combination of optogenetics with cell therapy has various potential clinical applications,^98^ but three major challenges need to be considered: tumorigenicity, immunogenicity, and heterogeneity. Sensitive in vitro systems such as the organ‐on‐a‐chip technique, have been developed to evaluate tumorigenicity, and factors that can reduce heterogeneity have been identified.^99^ Another challenge is to maintain the survival of transplanted cells for an extended period in the body cavity or in target organs. Encapsulation by using biomaterials such as hydrogels, ceramics, metals, and plastics, with spheres larger than 1.5 mm in diameter can ameliorate immune responses, promote survival post transplantation and maintain the activity of implantable engineered cells.^100^ Numerous clinical trials have been conducted to evaluate the use of encapsulated cells in treating human diseases, especially diabetes. Moreover, recent advances in material science, nanotechnology, and immunomodulation have led to promising techniques in cell‐based microencapsulation and microencapsulation.^101^, ^102^

Cell encapsulation requires intense investigation into how to provide a controlled and supportive environment for the maintenance of cell functions^103^ and to diminish immune rejection. Usually, the transplanted cells are protected from immune rejection by an artificial, semipermeable membrane, which potentially allows for transplantation (allo‐ or xenotransplantation) without the need for immunosuppression.^104^ This semipermeable membrane allows for normal insulin secretion in response to fluctuating blood glucose levels while maintaining cell viability through sequestration from the immune system and maintaining effective nutrient and waste exchange. Various materials such as alginate, polyethylene glycol, agarose, and cellulose have been applied in microencapsulation by virtue of their advantages in immunoisolation. However, those materials share common limitations, in which the transplanted cells cannot be retrieved in case of loss of function, adverse effects, or malignant transformation. Another challenge is to maintain cell survival, and it might face many obstacles, including dispersion, suboptimal oxygenation, and engraftment. Macro‐encapsulation systems that consist of membrane‐encased cells and devices with a planar or cylindrical geometry are able to provide a sustainable microenvironment for the cells and can also be retrieved in the case of dysfunction. Intravascular and extravascular systems are two major disposal pathways. The former connects the graft to systemic circulation but needs to avoid thromboembolic events, and the latter relies on new blood vessel formation at the host tissue–device interface before it starts to work. Both are designed as tubular or planar diffusion chambers that can be implanted in the peritoneal cavity, or subcutaneous tissues, in consideration of surgical procedure and minimal invasion.^103^

## 7 SMART DEVICES FOR OPTOGENETICS‐BASED CELL THERAPY AND BLOOD CONTROL

**FIGURE 2:**
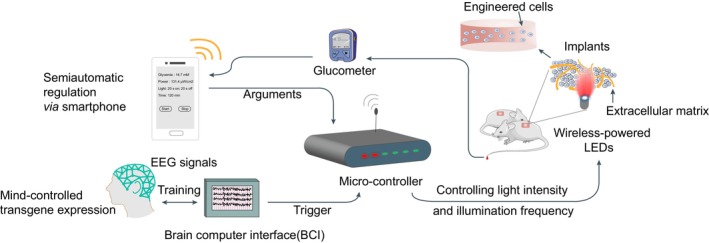
Development of smart devices for optogenetics‐based cell therapies. The mind‐controlled semiautomatic regulation of gene expression via smartphone. The brain‐computer interface (BCI) is established to read electroencephalogram (EEG) signals, where the brain‐wave activities are analyzed in a smartphone, transmitted via Bluetooth to the microcontroller. The microcontroller automatically integrates the reception, processing, and execution of these signals in a programmable manner to control the wireless‐powered light‐emitting diodes (LEDs) and their illumination intensity and frequency. Alternatively, the near‐infrared (NIR) emitting LEDs from the wireless‐powered optogenetic implant can be used to illuminate the engineered cells to produce insulin or glucagon‐like peptide‐1, which would diffuse to the blood through a semi‐permeable membrane. The blood glucose level can be continuously monitored by the glucometer, which sends real‐time information to the smartphone to adjust other parameters for optimal treatment.

Bioelectronics is a highly interdisciplinary subject, which requires new developments across the physical and life sciences.^105^ The incorporation of bioelectronics into optogenetics envisions novel medical devices that bridge optogenetics‐based therapies to clinical translational applications. Namely, these smart devices consist of three key components: blood‐sugar monitor, central controller, and implantable wireless‐powered LED light source.^69^, ^106^ The devices ultimately achieve closed‐loop regulatory mechanism, in which the central controller receives glycemia from the monitor and then sends appropriate parameters, such as the light intensity and illumination frequency, to the LED light source. The central controller functions as a central processor that is trained by repetitive machine learning algorithms based on previous regulatory data and can automatically make decisions according to current glycemia and body condition. It can also act as a receiver, or more precisely a mind‐controlled medical device, that reads commands from the mobile terminal (eg, smartphone) and then sends the right arguments to the stimulator according to the EEG of a diabetic patient (Figure 2).^107^

In 2014, Folcher et al first introduced a mind‐controlled transgene expression device.^107^ This study deciphered raw brain‐wave activities captured by an EEG headset and identified mental state‐specific electrical patterns as discrete meditation‐meter values. They designed an electro‐optogenetic interface for subjects, which assists them in maintaining the biofeedback‐derived meditation‐meter value in the specific threshold range after intentional training. Brain‐wave activities beyond the default threshold were supposed to send the command to the microcontroller and by which to further switches on a wireless‐powered illumination implant for photoactivation of diguanylate cyclase, a truncated phosphodiesterase domain‐deficient *R. sphaeroides* BphG1 variant. Similarly, the Ye group developed a SmartController device that harbors a 32‐bit embedded microprocessor unit (MPU) chip, which is responsible for the automatic integration of the reception, processing, and execution of electronic signals in a programmable manner.^46^ The chip converted the inputted high‐voltage alternating current and outputs low‐voltage direct current within the mainboard interior. The receiver translated wireless fidelity (Wi‐Fi) signals from an internal antenna into MPU‐compatible messages. A bluetooth‐active glucometer monitored blood glucose levels and sent the data to the controller, which further signaled to the corresponding far‐red light for appropriate illumination dosage. After implantation of GLP‐1‐secreting engineered cells (Bphs as the optogenetic transgene) into diabetic mice, they used the SmartController device to successfully achieve remote and semiautomatic regulation of glucose homeostasis. Moreover, the same group recently improved the illumination module and developed an implantable HydrogeLED.^56^ These coiled‐LEDs could be wirelessly charged by a custom‐designed electromagnetic emission circuit and emit far‐red light for Bphs activation. They also applied a flexible implantable piezoelectric nanogenerator to supply long‐term energy by converting biomechanical energy into electricity, which achieved a self‐powered optogenetic system.^108^ Furthermore, it has been shown that some optogenetic modules such as melanopsin and TtCBD could even be activated by the flashlight of the smartphone or the green light from a smartwatch.^69^

Collectively, these studies combine the unique capacity of electronic devices to read and generate digital signals with the high theranostic precision of biological cells.^109^ The future of automated insulin delivery technology integrating data‐driven algorithms and wearable micro‐medical devices can make the management of diseases more precise and personalized, especially greatly improving the treatment experience of patients with chronic diseases.

## 8 CONCLUSION AND OUTLOOK

Over the past few years, the applications of optogenetics have been massively extended. Because the field of gene and cell therapy is evolving rapidly, there is no doubt that optogenetics will be an alternative therapy for certain diseases if some regulations are cleared. In the past few years, studies have been focused on achieving high light penetration depth for in vivo applications, developing more sensitive and effective light responsive modules, and optimizing the biological safety for embedded systems. In addition, studies have been made to target other components in cells besides the regulation of insulin secretion itself, such as modulation of neuroendocrine pathways, insulin‐target tissues, and other diabetic complication‐susceptible tissues. However, optogenetics‐based therapy still faces many challenges in clinics, including the drawbacks of optogenetics itself and the challenges for diabetes management. First, optogenetics requires artificial optical stimulation, which raises the question of efficiency and safety. Most of the optogenetic modules are activated within the visible light spectrum with poor light penetration, and although this can be improved to some extent by using implanted UCNPs, micro‐LEDs, or intracellular bioluminescence, the safety and efficiency still need to be verified. Wireless implantable devices have been reported for light delivery, but many issues need to be taken into consideration, such as heat generation, invasive implantation, device longevity (power supply), and any induced immune responses.^110^ Therefore, the development of red‐shifted photoresponsive proteins is important, which would allow for deeper tissue penetration. Second, the optogenetic systems need the delivery of gene encoding light‐responsive proteins, and its safety, effectiveness, and especially use for cell‐specific gene targeting are required to be evaluated for clinical applications. Currently, the adeno‐associated viruses (AAVs) are preferred for gene delivery as to their safety, flexibility, and excellent tissue penetration properties. However, the low packaging capacity of AAVs (~ 4.5 kb) limit their application to express only small therapeutic genes. For transplantation of engineered cells, the limitations in cell encapsulation, such as leakage of engineered cells from the implant, immune rejection, and carcinogenesis, would induce new obstacles in clinical applications. Third, the ideal therapies should be able to mimic the physiological insulin secretion patterns in both basal and postprandial conditions, which include secretion amount, rate, and responsiveness to glucose. Preprandial insulin injection or antidiabetic administration could lower the blood glucose down to the rational range by controlling the drug dosage. However, optogenetics‐based cell therapy might face the challenge of releasing the appropriate dose of insulin, which is associated with the functionality of the optogenetic implants. It is difficult to establish stable and repeatable quantitative secretion of insulin by titrating the light illumination. Unbalanced insulin release would easily cause hypoglycemia or hyperglycemia in diabetes. However, the integration of bioelectronic devices with optogenetics would provide a feasible automatic therapeutic strategy, as the dosage regimen adjusts to the physiopathological changes, which would improve the quality of life of patients on long‐term prescriptions. Lastly, although efforts have been taken to ameliorate insulin resistance by using optogenetics to regulate insulin signaling or neuroendocrine activity, most of them lack long‐term experimental results and are absent of systemic therapeutic effects. T2DM is characterized by insulin resistance, hyperinsulinemia, and chronic inflammation. More accurate understanding of the pathophysiologic mechanism of the disease would be helpful to discover better therapeutic targets. Optogenetics sheds new light on the treatment of diabetes; meanwhile, other comprehensive therapies including lifestyle management and glycemic management to minimize long‐term complications are equally important. Modern technology, such as intermittently scanned glucose monitoring and continuous glucose monitoring, together with new drug therapies (eg, ultra‐fast insulins, sodium glucose transporter 2 inhibitors, and GLP‐1 receptor agonists), could help to change the landscape of glycemia management.^111^ However, these pharmacological approaches could either lead to irreversible phenotypes in the host cells or induce undesirable cytotoxicity and signaling cross‐talk due to drug off‐target or their pleiotropic effects.^112^ Compared with chemical drugs, engineered optogenetic modules usually have very specific targets, can be designed to be reversible, and can be activated or deactivated in a light‐controllable manner. The optogenetics‐based therapies have recently approved to be used in clinic to restore vision or hearing in patients. Similarly, we anticipate that optogenetics would have great potential in neuroendocrine regulation, in which the central or peripheral nervous systems can be directly targeted to improve glucose metabolism, which would not only stabilize blood glucose but also enhance β‐cell proliferation. In addition, optogenetic modules can be engineered to be expressed only in a specific cell type or targeted to a chosen metabolic pathway. By these means, it will facilitate elucidation of the pathogenesis of T2DM and would yield new therapeutic strategies. Furthermore, the development of closed‐loop and deep‐tissue optogenetic devices with safe gene delivery technology would further promote the translation of optogenetic therapy to the clinic in the future.

## CONFLICT OF INTEREST STATEMENT

The authors declare no competing interests. No human or animal studies involved or no ethical statement for the study.
